# Quality of antenatal care and its sociodemographic determinants: results of the 2015 Pelotas birth cohort, Brazil

**DOI:** 10.1186/s12913-021-07053-4

**Published:** 2021-10-09

**Authors:** Lina Sofia Morón-Duarte, Andrea Ramirez Varela, Andrea Dâmaso Bertoldi, Marlos R. Domingues, Fernando C. Wehrmeister, Mariangela Freitas Silveira

**Affiliations:** 1 Post-Graduate Program in Epidemiology, Federal University of Pelotas, Pelotas, RS Brazil; 2 Rua Marechal Deodoro 1160 – Centro, Pelotas, RS 96020-220 Brazil; 3 School of Medicine, Universidad de los Andes, Cra. 7 #116-5, Bogotá, Colombia 11001000; 4 Post-Graduate Program in Physical Education, Federal University of Pelotas, Pelotas, RS Brazil

**Keywords:** Antenatal care, Quality of health care, Health care inequality, Social determinants of health

## Abstract

**Background:**

Inadequate antenatal care (ANC) has been associated with adverse pregnancy outcomes. ANC quality is considered a key component of the right to health and a route to equity and dignity for women and their children. Although ANC coverage is relatively high in Brazil, there are revealed some health disparities when coverage is examined by socio-demographic determinants. In this study we evaluated ANC quality and its socio-demographic determinants using data from the 2015 Pelotas birth cohort, Rio Grande do Sul, Brazil.

**Methods:**

This study is part of the 2015 Pelotas population-based birth cohort (*n* = 3923 pregnant women) conducted in southern Brazil. ANC quality was assessed through 19 content and service utilization indicators recommended by the Brazilian Ministry of Health. Descriptive analyses and associations of each of the ANC indicators and independent variables were performed using the chi-square and linear trend test. ANC indicators were analyzed individually and aggregated as a score. Associations between ANC score quality and socio-demographic variables were assessed with ordinal regressions. Mediation analysis with G-computation was performed to estimate direct and indirect effect of mother’s level of education on ANC quality mediated by the number of consultations and timing of ANC initiation. Base and post confounders were included.

**Results:**

The results showed that except for breast examination, height measurement, tetanus toxoid vaccination and ANC starting at the first trimester, all ANC indicators showed more than 80% coverage during ANC visits.

In the adjusted analysis, inadequate quality ANC was associated with lower maternal education level, not having a partner, being multiparous, being attended by a private provider and by the same professional in all consultations. In the mediation analyses, 6.8% of the association between ANC quality and mother’s education was mediated by the trimester in which ANC started, while 12.8% was mediated by the number of ANC visits.

**Conclusions:**

ANC quality is associated with pregnant women’s socio-demographic characteristics. Significant efforts are needed to improve the quality of facility-based maternity care.

**Supplementary Information:**

The online version contains supplementary material available at 10.1186/s12913-021-07053-4.

## Background

Antenatal care (ANC) is the care given to pregnant women that is aimed at securing a safe pregnancy and a healthy baby. Inadequate prenatal care has been associated with adverse pregnancy outcomes. In 2015, about 303,000 women in the world died of pregnancy-related causes [1]. Health care quality during pregnancy and childbirth can prevent many of these deaths. According to the World Health Organization (WHO), nearly 3/4 of maternal deaths in poor countries are preventable, 26% with adequate prenatal care and 48% with increased access to quality obstetric care [1].

The different models of ANC routines (appropriate time to start consultations, periodicity, and the content of care) have been the subject of constant analysis due to the variability in practice across countries over time, especially in trying to set necessary minimum standards that are cost-effective to prevent maternal and child morbidity and mortality [2]. From this perspective, WHO has been developing initiatives to evaluate the effectiveness of different types of maternal health care models by proposing minimum packages for ANC [3] and has recently issued new comprehensive recommendations on routine ANC that have universal components applicable to all women. The recommendations are designed to be adaptable to each country’s context and to the characteristics of its populations to improve the quality of prenatal care, reduce the risk of stillbirth and pregnancy complications and provide women with positive experience in pregnancy [4–6]. Regarding the frequency of visits, the new guideline recommends a minimum of eight during pregnancy, emphasizing the importance of high-quality care during each contact, providing the proven effective procedures and interventions at each visit, and expanding the definition of ANC quality by considering content, service utilization and delivery indicators [4].

In parallel, it is necessary to consider social determinants such as maternal education level and family income that play an important role and have been consistently linked to the quality of antenatal care received by health services [7–9]. Evidence from several studies suggests that maternal education may also be associated with starting ANC early and the total number of ANC visits a woman makes during pregnancy [10–14]. The number of ANC visits defines the opportunity for receiving quality ANC; the more contact pregnant women have with health services, the greater the possibility of monitoring and evaluating their health status and receiving all procedures, exams and interventions included in the ANC package [4, 6]. We hypothesize that education level is associated with the quality of care directly and indirectly through its association with the number of ANC visits and starting ANC.

Accordingly, our objectives were 1) to evaluate ANC quality and its socio-demographic determinants using data from the 2015 Pelotas birth cohort, Rio Grande do Sul, Brazil; 2) to estimate the direct and indirect effects of maternal education on ANC quality, mediated by the number of visits and trimester of initiation of ANC.

## Methods

This study was based on the mothers of children eligible for the 2015 Pelotas Birth Cohort who were interviewed during pregnancy and/or at birth. The total number of eligible pregnant mothers was 4329 (in the case of multiple pregnancies only one record was kept for each mother). Fifty-nine records corresponded to multiple births, totaling 4270 mothers in the birth cohort. Of these mothers, 98 did not have ANC therefore they were excluded from the analysis (socio-demographic characteristics in Supplementary File 1), resulting in a final sample of 4172 mothers included in this study (249 did not have an antenatal card available at the time of interview) (Supplementary File 2).

Mothers were interviewed in the maternity ward a few hours after delivery and answered a standard prenatal questionnaire containing questions on ANC, as shown in Table 1. The interviewers were personnel trained by the researchers to conduct the interviews and had nothing to do with the ANC. At the end of the interview, the portable prenatal care card was photographed and subsequently transcribed twice by two trained research assistants into a final database built in Epi Info 6.04 (Centers for Disease Control and Prevention, Atlanta, USA). Additionally, a consistency analysis was performed by cross-checking the frequencies of the prenatal care card data and the data transcribed by the research assistant.

**Table 1:** Questionnaire items and the antenatal care card variables

Self-report questionnaire*	Antenatal care card^a*^
1. How many antenatal care visits did you have? 2. Did the doctor or nurse measure your weight? (yes/no) 3. Did the doctor or nurse measure your abdomen? (yes/no) 4. Did the doctor or nurse measure your blood pressure? (yes/no) 5. Did the doctor or nurse do the gynecological exam? (yes/no) 6. Did the doctor or nurse take cervical cancer prevention exam? (yes/no) 7. Did the doctor examine your breasts? (yes/no) 8. During your antenatal care, did you get the vaccine for tetanus toxoid or tetanus-diphtheria-acellular pertussis (Tdap)? (Yes, or already vaccinated/no)^b^ 9. Did the doctor give a medical prescription for anemia (iron)? (yes/no) ^c^ and 9.1 Did the doctor give a medical prescription for vitamins? (yes/no)	1. Number of antenatal care visits report 2. Weight measurement 3. Symphysis-fundal height measurement 4. Blood pressure measurement 5. Gynecological exam 6. Cervical cancer screening test 7. Breasts exam 8. Record of vaccine for tetanus toxoid or tetanus-diphtheria-acellular pertussis (Tdap)^b^ 9. Record of iron supplements prescription ^c^ and record of vitamins / folic acid prescription 10. Height, 11. Fetal heart sounds, 12. Fasting blood glucose test, 13. ABO-Rh test, 14. Hemoglobin test, 15. Urine test, 16. Human immunodeficiency virus (HIV) test, 17. Syphilis test 18. Ultrasound scan 19. Start ANCat the first trimester

^a^Variables were recategorized as “yes” if they had at least one record on the card and “no” when there was no record

^b^Any doses, booster, or already vaccinated were considered as: “yes”

^c^This question was coupled with the question about medical vitamins/ folic acid prescription

^*^For number questions of the 1 to 9.1 were combined both sources and the number questions 10 to 19 were used only of the antenatal card

The questionnaire used during the perinatal time of the 2015 Pelotas Birth Cohort is available at: http://epidemio-ufpel.org.br/site/content/coorte_2015/questionarios.php. More detailed information on the methods and follow-up visits within the cohort is provided elsewhere [15].

### Outcome variables

Information on the content and health service utilization ANC indicators was taken from the prenatal card and maternal self-report questionnaire (Table 1). Only the prenatal card was used to obtain information for the following indicators, not covered by the questionnaire: mother’s height, fetal heart sounds, fasting blood glucose test, ABO-Rh test, hemoglobin test, urine test, human immunodeficiency virus (HIV) test, Syphilis test, ultrasound scan, and start ANC at the first trimester. For the remaining indicators (weight, blood pressure, symphysis fundal height, gynecological exam; breast exam; cervical cancer screening test; tetanus toxoid vaccination; iron and folic acid supplements; and number of visits) we used both sources, combining them by designating those that had information as done, and those that did not as not done. This means that if there was at least one “yes” record (in the antenatal card or the self-report questionnaire) it was considered done, otherwise it was considered not done (Table 1).

The number of antenatal care visits was categorized (≤5 or ≥ 6 visits) and the start of ANC at the first trimester was estimated with the date of birth of the child and the date of the first visit recorded in the portfolio. Each indicator was evaluated as binary (yes / no).

ANC quality was estimated by constructing a score based on the 19 previously listed ANC content and health service utilization indicators, expressing whether women received them during any of their ANC visits. One point was given for each of the procedures listed above, resulting in an additive score with a score ranging from 1 to 19. Subsequently, for multivariate analysis, the score was categorized considering its distribution: inadequate quality ≤15 points, moderate quality ≥16 to ≤17 points, and ≥ 18 points, as adequate quality. For our analyses, we distinguished between women who received all (18 to 19) components of care (adequate quality) and the women who received 15 or fewer (inadequate quality). The decision to define the dependent variable in this manner is based on the premise that all the ≥18 components are essential for quality pregnancy care [6].

### Independent variables

Demographic and socioeconomic characteristics were collected during the perinatal interview. Independent variables included mother’s age in complete years, categorized as ≤19 years, 20 to 29 years, 30 to 39 years and ≥ 40 years; mother’s education in complete years, expressed in four categories: 0–4 years, 5–8 years, 9–11 years and ≥ 12 years; maternal marital status, expressed as living with a partner or not; self-reported skin color, containing the options white, black/brown, yellow and indigenous, the last two being categorized in “others”; family income in quintiles (Q1 being the poorest and Q5 the richest); hypertension and/or diabetes during pregnancy (yes/no); smoking during pregnancy (yes/no); alcohol use during pregnancy (yes/no); parity (primiparous/≥2 children); type of health service provider during pregnancy (public/private); and whether the prenatal care professional was always the same or not.

### Analysis

In addition to descriptive analysis of the studied sample, the frequencies of each of the ANC indicators/components were calculated according to sociodemographic, reproductive and health service characteristics using the chi-square test for heterogeneity (nominal variables) and linear trend test (ordinal variables).

Associations between ANC content quality score categories and independent variables were assessed using bivariate and multivariate ordinal logistic regression analysis. The proportionality of the odds ratio was tested by the Brant test, which showed no violation in its assumption (*p* values > 0.05). For multivariate analysis, adjustments were made following the conceptual framework [16] (described in Supplementary File 3). At this stage, all the variables that showed a statistically significant association (*p* ≤ 0.2) were maintained in the analysis model. First, we include the first level -socio-demographic-, then the second level -reproductive and maternal characteristics- and finally the third level -health service-. Associations of all variables with outcome (from distal to proximal level) were tested.

Data were analyzed using the statistical package Stata, version 15. (Stata Corporation, College Station, Texas, United States).

### Mediation analysis

For the mediation analysis, the ANC content quality score was used continuously and only with indicators related to a physical exam, the diagnostic approach used and preventive measures, excluding the antenatal care services utilization assessment indicators (number of visits, and start ANC at the first trimester). The latter were evaluated as mediators.

For the mediation analysis, G-computation (bootstrap replications: 10,000) through Monte Carlo simulation was performed via the G-formula [17] command in Stata 15 to estimate the direct and indirect effect of the level of education on the quality of ANC content mediated by the number of consultations and ANC initiation trimester. The base confounder and post confounder variables were considered for each mediation analysis specified above. The analysis schemes for greater comprehension are presented in Supplementary File 4.

The antenatal follow-up of the 2015 Pelotas (Brazil) Birth Cohort Study was approved by the Higher School of Physical Education Research Ethics Committee from the Federal University of Pelotas under the protocol 522.064. Women indicated their agreement in participating in the study by signing an informed consent form.

## Results

Table 2 presents the sample distribution according to the variables studied. Of the 4271 mothers, the majority was white (71.8%), between 20 and 39 years old (82.6%), had between 9 and 12 years of schooling (66.1%), was primiparous (50.1%), had a partner (86.2%) and was attended by a private provider (55.2%).

**Table 2:** Distribution of maternal characteristics included in the present analysis of the 2015 Pelotas birth cohort, Rio Grande do Sul, Brazil

Characteristics	Women that had antenatal care N (%) ***N*** = 4172^a^
**Age (years)**	
≤ 19	602 (14.4)
20–29	1.982 (47.5)
30–39	1.466 (35.1)
≥ 40	122 (3.0)
**Maternal education (complete years of schooling)**	
0–4	365 (8.8)
5–8	1.050 (25.1)
9–11	1.447 (34.7)
12 +	1.310 (31.4)
**Marital status**	
Without partner	575 (13.8)
With partner	3.597 (86.2)
**Skin color**	
White	2.954 (70.8)
Black/brown	1.187 (28.5)
Other	31 (0.7)
**Family income (quintiles)**	
Lowest/first	813 (19.5)
Second	827 (19.8)
Third	846 (20.3)
Fourth	844 (20.2)
Highest/fifth	841 (20.2)
**Parity**	
Primiparous	2.088 (50.1)
≥ 2 children	2.083 (49.9)
**Diseases during pregnancy (high blood pressure and/or diabetes**^b^**)**	
Yes	1.279 (30.7)
**Smoking during pregnancy**	
Yes	661 (15.9)
**Alcohol use during pregnancy**	
Yes	302 (7.2)
**Type of health care provider**	
Public	1.389 (44.8)
Private	1.712 (55.2)
**The same professional performed the ANC**	
Yes	2.221 (53.2)

^a^The total of some variables does not sum to 4172 because of missing data

^b^The prevalence of high blood pressure was of 25.5% and for diabetes was of 8.6%

As shown in Table 3, it was observed that for the indicators related to the physical examination, 100, 99.9, and 99.8% of women reported having received blood pressure, weight, and uterine height measurements, respectively, in addition to 89.1% of them having undergone gynecological examination. Breast examination and height measurement were the least frequent (54.1 and 38.3%, respectively). Indicators referring to diagnostic/laboratory tests had good coverage, all equal to or above 90%. Regarding preventive measures, 91.7% of mothers received iron, folic acid or vitamin supplementation, and 76.1% received tetanus toxoid vaccination. Additionally, 55.7% of women began ANC at the first trimester and 86.2% had more than six ANC visits (Table 3). Finally, significant differences were observed in the proportion of ANC indicators covered according to maternal characteristics. These included lower coverage in younger women, those with fewer years of education, of low income, who reported having no partner, who self-identified as non-white, were multiparous, displayed risk behaviors during pregnancy (smoking/alcohol consumption), and were attended by the same health professional during ANC visits (Table 3).

**Table 3:** Distribution of indicators of antenatal care according to maternal characteristics of the 2015 Pelotas birth cohort, Rio Grande do Sul, Brazil

Characteristics	Physical exam							Diagnostic approach								Preventive measures		Antenatal care services utilization assessment	
	Weight measurement %	Height measurement %	Blood pressure measurement %	Symphysis-fundal height measurement %	Fetal heart sounds measurement %	Gynaecological exam %	Breast exam %	Cervical cancer screening test %	Fasting blood glucose test %	ABO-Rh %	Haemoglobin test %	Urine test %	Human immunodeficiency virus (HIV) test %	Syphilis test %	Ultrasound scan %	Tetanus toxoid vaccination %	Iron/ folic acid/vitamins supplements %	Start ANC at first trimester %	N° Visit ≥ 6%
**Age (years)**	0.524*	< 0.001*	–	0.314*	0.012*	< 0.001*	< 0.001*	< 0.001*	0.499*	0.143*	0.294*	0.330*	0.355*	0.981*	0.001*	0.190*	0.962*	< 0.001*	< 0.001*
≤ 19	100.0	55.0	100.0	100.0	93.8	84.1	49.5	51.5	91.5	91.2	90.3	88.5	89.9	90.8	89.2	78.4	92.0	40.5	74.1
20–29	99.9	41.0	100.0	99.8	94.1	89.0	51.2	68.1	91.3	89.7	92.0	90.7	91.1	91.4	90.8	75.0	91.5	56.3	86.7
30–39	99.9	28.9	100.0	99.7	92.2	91.0	59.2	73.8	91.8	89.6	91.7	90.3	91.6	91.2	93.8	78.2	91.9	61.6	90.1
≥ 40	100.0	24.4	100.0	100.0	87.8	93.4	60.7	72.9	93.9	85.2	93.9	91.3	90.4	90.4	90.4	83.6	91.8	50.5	91.8
**Maternal education**	0.062*	< 0.001*	**–**	0.771*	0.004*	< 0.001*	< 0.001*	< 0.001*	0.003*	0.793*	0.017*	0.005*	0.002*	0.065*	< 0.001*	0.292*	0.021*	**< 0.001**	< 0.001*
0–4	99.5	66.5	100.0	99.7	94.4	79.7	46.0	61.1	87.5	88.7	88.1	84.9	86.9	86.9	82.2	75.6	92.1	43.2	69.9
5–8	99.9	54.7	100.0	99.7	94.0	82.9	48.0	60.1	90.7	89.6	90.9	89.5	89.5	90.8	89.3	77.1	90.1	46.7	78.3
9–11	100.0	39.9	100.0	99.8	94.4	90.2	51.2	69.4	92.3	91.0	92.7	91.6	92.5	92.8	93.1	75.7	91.2	57.6	88.0
12 +	99.9	15.3	100.0	99.8	90.9	95.3	63.2	74.3	92.7	88.7	92.3	90.8	91.7	91.0	94.4	78.4	93.6	64.4	95.2
**Marital status**	0.515	**< 0.001**	–	0.206	0.213	**0.044**	**0.032**	**< 0.001**	**0.007**	0.159	**0.032**	**0.001**	**0.002**	**0.031**	**< 0.001**	0.166	0.062	**< 0.001**	**< 0.001**
Without partner	99.8	48.8	100.0	100.0	92.0	86.6	49.9	57.0	88.6	88.0	89.4	86.4	87.5	88.8	87.7	74.6	89.8	36.2	71.7
With partner	99.9	36.6	100.0	99.7	93.4	89.4	54.7	69.6	92.1	90.0	92.1	90.9	91.6	91.6	92.2	77.2	92.1	58.7	88.6
**Skin color**	0.629	**< 0.001**	–	0.959	0.151	**0.005**	0.052	**0.001**	0.772	0.751	0.572	0.697	0.529	0.799	**< 0.001**	**0.006**	0.606	**< 0.001**	**< 0.001**
White	99.9	32.4	100.0	99.8	93.0	90.1	55.0	69.5	91.8	89.9	92.0	90.0	91.3	91.4	93.3	76.0	91.7	59.8	89.1
Black/brown	99.8	53.2	100.0	99.8	94.1	88.6	52.1	63.8	91.2	89.4	91.0	90.8	90.3	91.8	87.4	79.5	92.0	45.9	79.4
Other	100.0	37.9	100.0	100.0	86.2	87.1	38.7	64.5	89.7	93.1	93.1	89.7	93.1	93.1	93.1	61.3	87.1	30.8	77.4
**Family income (quintiles)**	0.149*	< 0.001*	–	0.476*	0.004*	< 0.001*	< 0.001*	< 0.001*	0.188	0.047*	0.385*	0.269*	0.084*	0.486*	< 0.001*	0.392*	0.038*	< 0.001*	< 0.001*
Lowest/first	99.8	56.9	100.0	99.9	95.1	83.4	49.8	62.0	89.5	90.8	89.6	88.0	88.1	89.3	86.6	76.5	90.7	47.1	74.7
Second	99.9	52.7	100.0	99.6	93.6	85.3	52.4	64.8	93.0	90.6	93.5	90.7	92.2	92.5	90.6	75.9	90.3	52.0	82.5
Third	100.0	39.8	100.0	99.5	93.1	89.2	50.0	67.0	91.5	90.3	91.8	92.1	91.9	91.6	92.6	78.1	92.6	54.2	87.7
Fourth	99.9	29.6	100.0	99.8	92.9	91.1	51.1	68.3	91.7	89.0	91.8	90.2	91.5	91.7	94.3	74.5	92.2	59.8	90.2
Highest/fifth	100.0	12.8	100.0	100.0	91.3	96.0	66.9	76.9	92.3	88.1	91.9	90.2	91.3	90.8	93.8	79.2	93.9	65.6	95.7
**Parity**	0.998	**< 0.001**	–	0.524	0.380	**0.014**	**0.037**	**0.008**	**0.026**	0.220	0.109	0.213	**0.026**	**0.010**	**0.006**	**< 0.001**	**0.001**	**< 0.001**	**< 0.001**
Primiparous	99.9	32.2	100.0	99.8	92.9	90.2	55.7	65.9	92.6	90.3	92.4	90.8	92.1	92.4	92.8	80.9	93.1	58.7	89.1
≥ 2 children	99.9	44.5	100.0	99.7	93.6	87.9	52.4	69.8	90.6	89.2	91.0	89.7	90.0	90.1	90.4	72.8	90.4	52.7	83.3
**Diseases during pregnancy (high blood pressure and/or diabetes)**	0.806	**0.005**	–	0.521	0.817	0.287	**0.029**	0.994	0.988	0.466	0.858	0.927	0.515	0.673	0.981	0.482	**0.010**	0.752	0.768
Yes	99.9	41.5	100.0	99.7	93.1	88.3	51.5	67.9	91.6	90.3	91.6	90.2	90.6	91.5	91.6	77.6	90.1	56.1	86.0
No	99.9	36.8	100.0	99.8	93.3	89.4	55.2	67.8	91.6	89.5	91.8	90.3	91.3	91.1	91.6	76.6	92.5	55.5	86.4
**Smoking during pregnancy**	0.616	**< 0.001**	**–**	0.719	0.152	**< 0.001**	**< 0.001**	**0.002**	**< 0.001**	0.323	**0.007**	**0.001**	**0.001**	**0.020**	**< 0.001**	0.183	0.106	**< 0.001**	**< 0.001**
Yes	99.9	58.0	100.0	99.7	94.6	83.1	46.8	62.8	87.5	88.6	89.0	86.5	87.5	88.8	85.3	74.9	90.2	47.4	75.9
No	99.9	34.7	100.0	99.8	93.0	90.2	55.4	68.8	92.3	90.0	92.2	90.9	91.7	91.7	92.8	77.3	92.1	57.2	88.2
**Alcohol use during pregnancy**	0.576	0.067	–	0.376	0.155	0.834	0.053	**0.016**	0.959	0.578	0.671	0.691	0.777	0.975	**0.027**	0.652	0.503	**< 0.001**	**< 0.001**
Yes	100.0	43.5	100.0	100.0	95.3	89.4	48.7	61.6	91.7	88.8	92.4	90.9	90.6	91.3	88.0	75.8	90.7	40.3	77.5
No	99.9	38.0	100.0	99.8	93.1	89.0	54.5	68.3	91.6	89.8	91.7	90.2	91.1	91.2	91.9	77.0	91.8	56.8	87.1
**Type of health care provider**	**–**	**< 0.001**	**–**	0.572	**< 0.001**	**< 0.001**	**< 0.001**	**< 0.001**	0.759	**0.008**	0.399	0.340	0.236	**0.025**	**< 0.001**	**0.001**	0.994	**< 0.001**	**< 0.001**
Public	100.0	65.5	100.0	99.9	95.5	86.2	50.7	64.6	92.7	92.1	93.4	92.1	92.9	93.2	91.5	81.1	92.2	55.3	86.6
Private	100.0	15.4	100.0	99.8	91.6	94.4	57.6	73.7	92.4	89.3	92.6	91.2	91.7	91.0	95.3	76.3	92.2	63.0	93.2
**The same professional performed the ANC**	0.897	**< 0.001**	–	0.668	0.187	**< 0.001**	0.275	**< 0.001**	0.451	**0.024**	0.195	0.910	0.689	0.252	0.252	**< 0.001**	**0.022**	**< 0.001**	**0.034**
Yes	99.9	28.4	100.0	99.7	92.7	86.4	54.8	65.4	91.3	88.7	91.2	90.2	91.2	90.7	92.1	74.6	90.8	59.7	87.3
No	99.9	49.4	100.0	99.8	93.8	92.1	53.2	70.6	92.0	90.9	92.3	90.3	90.9	91.8	91.1	79.4	92.8	51.2	85.0
Total	99.9	38.3	100.0	99.8	93.2	89.1	54.1	67.9	91.6	89.8	91.7	90.3	91.1	91.2	91.6	76.9	91.7	55.7	86.2

*p-value for linear trend

Supplementary File 5 presents the frequency of the ANC content quality score according to the independent variables. It was observed that 35.0% of women were categorized as having received inadequate ANC quality (≤ 15 points), 40.9% moderate ANC quality (≥16 to ≤17 points), and 24.1% adequate ANC quality (≥18 to 19 points). Table 4 presents the unadjusted and adjusted analyses of the ANC quality score according to independent variables. After adjusting for confounding factors, women with fewer years of education, no partner and multiparous women had lower odds of having received adequate ANC. For the type of health service provider, it was observed that women attended by a public provider had 1.49 (95% CI, 1.27 to 1.76) greater odds of having received adequate ANC than both the moderate and inadequate ANC categories combined, given that all the other variables in the model were held constant. When women received prenatal care by different health professional, the odds of adequate ANC were 1.58 (95% CI, 1.37 to 1.81) times greater in relation to the combined moderate and inadequate ANC categories, given that all the other variables in the model were held constant.

**Table 4:** Crude and adjusted odds ratios of the Antenatal care content quality score (in categories^a^) according to the independent variables, using ordinal regression

Level	Characteristics	Antenatal care content quality score (in categories)			
		Crude OR	***p*** -value	Adjusted OR	***p*** -value
**1**	**Age (years)**		**< 0.001**		**0.001**
	≤19	1		1	
	20–29	1.36 (1.15–1.62)		1.32 (1.09–1.60)	
	30–39	1.51 (1.26–1.80)		1.55 (1.24–1.94)	
	≥40	1.21 (0.85–1.73)		1.34 (0.91–1.96)	
	**Maternal education (complete years of schooling)**		**< 0.001**		**0.018**
	0–4	0.66 (0.53–0.82)		0.79 (0.61–1.02)	
	5–8	0.76 (0.66–0.89)		0.93 (0.77–1.13)	
	9–11	0.93 (0.81–1.07)		1.11 (0.95–1.30)	
	12 +	1		1	
	**Marital status**		**< 0.001**		**< 0.001**
	Without partner	0.54 (0.46–0.65)		0.57 (0.48–0.68)	
	With partner	1		1	
	**Skin color**		**0.025**		0.167
	White	1		1	
	Black/brown	0.89 (0.78–1.01)		1.00 (0.87–1.14)	
	Other	0.48 (0.24–0.94)		0.51 (0.87–1.02)	
	**Family income (quintiles)**^a^		0.066		0.290
	Lowest/first	0.77 (0.64–0.92)		1.08 (0.87–1.34)	
	Second	0.91 (0.76–1.09)		1.15 (0.94–1.41)	
	Third	0.93 (0.78–1.11)		1.04 (0.85–1.26)	
	Fourth	0.88 (0.74–1.05)		0.93 (0.77–1.12)	
	Highest/fifth	1		1	
	**Parity**		**0.008**		**< 0.001**
	Primiparous	1		1	
	≥ 2 children	0.86 (0.76–0.96)		0.77 (0.67–0.88)	
**2**	**Diseases during pregnancy (high blood pressure and/or diabetes)**		0.731	–	–
	Yes	0.97 (0.86–1.10)		–	
	No	1		–	
	**Smoking during pregnancy**		**< 0.001**		0.098
	Yes	0.74 (0.63–0.87)		0.86 (0.72–1.02)	
	No	1		1	
	**Alcohol use during pregnancy**		**0.013**		0.181
	Yes	0.76 (0.61–0.94)		0.86 (0.68–1.07)	
	No	1		1	
**3**	**Type of health care provider**		**< 0.001**		**< 0.001**
	Private	1		1	
	Public	1.29 (1.13–1.47)		1.49 (1.27–1.76)	
	**The same professional performed the ANC**		**< 0.001**		**< 0.001**
	Yes	1		1	
	No	1.37 (1.22–153)		1.58 (1.37–1.81)	

^a^1 = Inadequate quality ≤15 points, 2 = moderate quality ≥16 to ≤17 points, and 3 = ≥ 18 points, as adequate quality

**Fig. 1:**
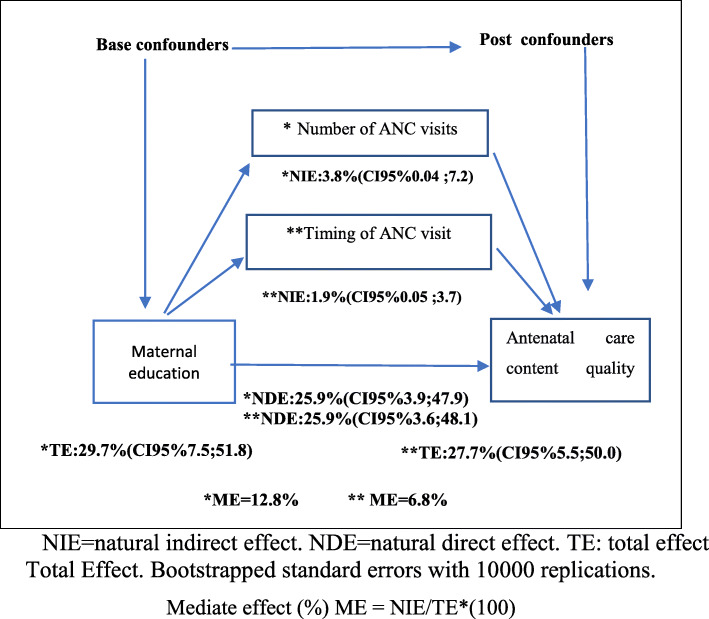
Direct acyclic graph of the association between maternal education and care content quality score in the 2015 Pelotas birth cohort, Rio Grande do Sul, Brazil. NIE = natural indirect effect. NDE = natural direct effect. TE: total effect. Total Effect. Bootstrapped standard errors with 10,000 replications. Mediate effect (%) ME = NIE/TE*(100)

In the mediation analyses, 6.8% of the association between ANC quality and mother’s education was mediated by the timing of ANC visits, and 12.8% was mediated by the number of ANC visits (Fig. 1).

## Discussion

Evaluation of ANC quality in terms of coverage has been described in the literature [18–21]. However, this approach is restricted and focuses on access to services only. Evaluating ANC quality using the content of ANC provided to pregnant women allows for a comprehensive view of the quality of care, contributing to the identification of the most vulnerable groups that are not receiving all procedures, exams and interventions included in the ANC package.

Our findings suggest that ANC indicators should be analyzed both individually and jointly (scores) because it allows for better evaluation of ANC coverage and quality. This is important for identifying specific and aggregated patterns of ANC indicators in accordance with the socio-demographic determinants studied.

### Specific patterns of ANC indicators and their sociodemographic determinants

When we evaluated the associations of each ANC indicator individually and studied the determinants, it was generally observed that women with higher socio-demographic vulnerabilities had a significantly lower proportion of ANC indicators than the rest. This situation reflects those inequalities in the utilization of ANC persist, directly affecting its quality. However, some of these associations disappeared when we used the constructed score to assess ANC quality in multivariate analysis.

### Socio-demographic determinants of ANC quality assessed using a score

We examined the relationship between socio-demographic determinants and ANC quality, defined in this paper by combining (score) key components performed during ANC. Only 6.5% of mothers received all 19 ANC components evaluated. On average, mothers received 15.9 items (minimum 3, maximum 19). Our findings suggest that women with a higher level of education, who are with a partner, who are primiparous, who sought care in the public sector and who were attended by different health professionals during their ANC have a positive association with adequate ANC quality, even after adjustment for confounder.

The results of our study corroborate those already found by previous investigations that observed an association between more highly educated women and adequate quality. This may indicate that educated women have more knowledge about the necessary procedures to be received during ANC, thereby increasing their chances of receiving qualified care and empowering them to demand access to such care. Several studies have documented greater confidence in women to act on their own health and greater awareness of the advantage of using health services during pregnancy or childbirth among women with higher levels of education, as compared to women with less schooling [8, 22–25].

On the other hand, it is possible that health professionals discriminate against poorly educated women by not providing comprehensive information on pregnancy care and not ordering all necessary examinations and tests or preventive measures, which are included in the ANC. Low maternal health services utilization among disadvantaged women has been associated with a perception of stigma and discrimination in the healthcare environment [26, 27].

We also found that pregnant women with partners are more likely to receive ANC of adequate quality. These findings were corroborated by a study that showed how spouses or partners can influence women to participate in ANC [28], producing better results by ensuring that women attend their ANC consultations and receive quality services. Yet, these results also contrast with those reported by other authors [29].

We also found significant differences regarding parity. Multiparous women received lower quality care than nulliparous women. Evidence has shown that this association can be explained by a late recognition of pregnancy [30], low risk appreciation due to experiences in previous pregnancies and deliveries [31, 32], and supply side obstacles that contribute to this problem [33]. Another important finding was the positive association that remained after adjusting for the type of public health service provider and ANC quality. This finding indicates an improvement in ANC service provision in this sector of the municipality of Pelotas, in relation to a previous study which reported gaps in ANC quality with limited follow-up of ANC content indicators and differences in the quality of care received in the public and private sectors [34].

We observed that when women had the opportunity to be seen by different health professionals during ANC (nurses, gynecologists), they were more likely to have received adequate ANC. One possible explanation is that women in contact with different providers have a greater opportunity to be evaluated differently and to receive all ANC procedures and interventions. These findings differ from those of other authors, who have reported that pregnant women attended by the same health professional during prenatal care may have better results due to bonding, the development of trust with the provider, and better monitoring of pregnancy [33]. Other studies have also found the fragmentation of care through consultations by different providers to be associated with poor prenatal care quality [35].

### ANC quality: mediation analysis

When we examined the mediating role of the number of ANC appointments and the time of initiation of ANC in the relationship between educational attainment and the quality of care received during pregnancy, we observed that only a small fraction of the total effect was mediated by these indicators of utilization and provision of health services. While these results are significant, it is necessary not to lose sight of the fact that there are multiple factors in a health system (distribution of health services, qualification of the health professional; infrastructure; availability of medications and equipment) and the application of sociocultural protocols/guidelines that may prevent even the most educated women from having access to quality ANC. Nonetheless, the purpose of mediation analysis in this paper was to allow better understanding of the complex relationship between education and ANC quality. The strong direct effect of the level of education on quality of care persists after controlling for confounding variables. This means that even when women access certain types of care on a timely basis (first trimester) and on a regular basis (multiple contacts or visits), their level of education still makes a difference in the quality of care they receive. This can be explained in part by the fact that women with higher income are generally the most educated, have easier access to health services and may demand with greater empowerment the services needed during pregnancy, all of which contribute to better quality care [23, 36].

### Strengths and limitations

This study has several strengths. First, the range of ANC care we reviewed included several indicators and were analyzed both in a disaggregated manner and jointly, despite not being exhaustive. Secondly, the study uses a population-based sample of women who gave birth during 2015 in the municipality of Pelotas, having high validity. Moreover, we included all women who gave birth, including those with stillbirths, reducing the chances of excluding women who received the worst care. Another strength is that we believe that reverse causation is a minor problem in our study, as the quality of ANC is highly unlikely to determine years of schooling, family income, and skin color, for example. The opposite is more plausible, thus increasing confidence in causal inference based on the temporal ordering of events.

Despite the important findings presented here, some limitations of the study require mention. First, the ANC quality measure captures only some aspects of the process and slightly less of the dimensions related to structure. Our analysis is useful for assessing whether women have received essential ANC services but does not capture women’s experience with the health system, nor the qualification and experience of the ANC providers. Several authors have concluded that it is important to include domains related to women’s care experience, empowerment, and autonomy to assess the quality of health service delivery. The client’s perception of the ANC quality received is influenced by having a qualified, respectful, informed provider, with availability of health technologies and the timely provision of services [37–39]. Therefore, the quality measure presented here is incomplete. However, data on these domains are often not collected due to the difficulty in their evaluation, explained by the lack of validated instruments that can be used in research questionnaires [40, 41].

Another limitation is that our ANC quality measure was restricted to the list of ANC indicators they received at least once during pregnancy, limiting the discrimination between different quality levels, as well as the ability to determine whether women have been properly followed-up with and directed during pregnancy. However, we consider that such limitations do not undermine our findings but suggest that the high-quality ANC score should be carefully analyzed, and that the magnitude of disparities in quality is potentially greater than what was shown in this analysis. Thirdly, we used two sources (questionnaire and prenatal card) of information to measure ANC indicators, which increased the chance of having higher ANC content and service utilization indicators scores, thus possibly overestimating the adequacy level of ANC quality. Moreover, while restricting the sample to women who had ANC (necessary for analysis) may reduce the generalization of findings, most factors that predict the quality of ANC were identified in women who had at least one ANC consultation. Recall and social desirability bias are also possible limitations, since the study utilized women’s self-reported information as one of its sources of data. Although the recall period was cut short, we believe that the accuracy of the reporting of care received was not affected. Regarding social desirability bias, women can report that they received services because they know they should receive them, which may lead to an overestimation of the quality of care.

Future research should focus on identifying the perceptions of ANC users about health service providers. Additionally, it is necessary to carry out comprehensive evaluations that include indicators of structure, processes, and results. On the other hand, it is necessary to create a score that evaluates the quality of the ANC based on all the components recommended by the WHO to monitor, evaluate, and compare the quality of ANC at a global level. It is necessary to use a conceptual and operational model for this evaluation. We consider that the model established by the WHO can be used to standardize the quality evaluation of ANC.

## Conclusions

The socio-demographic characteristics of pregnant women are related to the quality of ANC, and our findings support the fact that women with less education, without a partner, pregnant women with ≥2 children, attended by a private provider and by the same professional in all healthcare appointments are factors associated with a lower chance of adequate quality of ANC. Another important finding of our study is that the indicators of service utilization (start at the first ANC visit and number of ANC visits) are mediators of the association between maternal education level and ANC quality, but the mediated proportion we found indicates that efforts to improve the quality of ANC depend on more factors than solely early initiation of ANC and the number of consultations. Indeed, there are other factors affecting the quality of prenatal care a woman receives.

We recommend making efforts to improve ANC for disadvantaged women by focusing on removing structural barriers to access and strengthening the technical and interpersonal skills of providers. Such efforts should also seek to empower disadvantaged women to insist on quality prenatal care. In addition, special attention should be paid to women with low economic status and low educational level. The early age group and the late age group among women in the childbearing age group should receive special attention for services.

## Supplementary Information

**Additional file 1 Supplementary file 1.** Distribution of maternal characteristics who did not attend or receive antenatal care of the 2015 Pelotas birth cohort, Rio Grande do Sul, Brazil.

**Additional file 2 Supplementary file 2**. Figure flowchart sample.

**Additional file 3.** Supplementary File 3 – Figure conceptual framework for the analysis of factors associated with the quality ANC in the 2015 Birth Cohort in Pelotas, Rio Grande do Sul, Brazil.

**Additional file 4 Supplementary file 4.** Diagrams of conceptual framework of the effects of maternal education on the quality of antenatal care received.

**Additional file 5 Supplementary file 5**. Frequency of the Antenatal care content quality score in categories according to the independent variables.

## Data Availability

The data that support the findings of this study are available from the 2015 Pelotas (Brazil) Birth Cohort, but restrictions apply to the availability of these data, which were used under license for the current study, and so are not publicly available. Data are, however, available from the “Centro de Pesquisas Epidemiológicas (CPE) of Pelotas, RS, Brazil” on request by the publications committee upon reasonable request at [cpublicacoes.coortespelotas@gmail.com].

## Abbreviations

ANC: Antenatal care
WHO: World Health Organization
HIV: Human Immunodeficiency Virus- test
CI: Confidence interval
OR: Odds Ratio
NDE: Natural Direct Effect
NIE: Natural Indirect Effect
TE: Total Effect
