# Supplementary material for: Quality of antenatal care and its sociodemographic determinants: results of the 2015 Pelotas birth cohort, Brazil
Source: BMC Health Serv Res. 2021 Oct 9;21:1070. doi: 10.1186/s12913-021-07053-4 (PMC8501641; doi:10.1186/s12913-021-07053-4)
Supplement: Supplementary file 1 — Additional file 1 Supplementary file 1. Distribution of maternal characteristics who did not attend or receive antenatal care of the 2015 Pelotas birth cohort, Rio Grande do Sul, Brazil. [file 12913_2021_7053_MOESM1_ESM.docx]

| **Supplementary File 1. Distribution of maternal characteristics who did not attend or receive antenatal care of the 2015 Pelotas birth cohort, Rio Grande do Sul, Brazil.** | |
| --- | --- |
| **Characteristics** | **N (%) N= 98*** |
| **Age (years)** |  |
| ≤19 | 29(26.9) |
| 20-29 | 43(44.3) |
| 30-39 | 24(24.7) |
| ≥40 | 4(4.1) |
| **Maternal education (complete years of schooling)** |  |
| 0-4 | 29 (29.9) |
| 5-8 | 48(49.5) |
| 9-11 | 16(16.5) |
| 12 + | 4(4.1) |
| **Marital status** |  |
| Without partner | 44(45.4) |
| With partner | 53(54.6) |
| **Skin color** |  |
| White | 51(52.6) |
| Black/brown | 46(47.4) |
| Other | --- |
| **Family income (quintiles)** |  |
| Lowest/first | 45(46.4) |
| Second | 27(27.8) |
| Third | 11(11.3) |
| Fourth | 13(13.4) |
| Highest/fifth | 1(1.0) |
| **Diseases during pregnancy (high blood pressure and/or diabetes)** |  |
| Yes | 20(20.4) |
| **Smoking during pregnancy** |  |
| Yes | 53(55.2) |
| **Alcohol use during pregnancy** |  |
| Yes | 18(18.6) |
| **Parity** |  |
| Primiparous | 26(26.8) |
| ≥ 2 children | 71(73.2) |
| *The total of some variables does not sum to 98 because of missing data. | |
