# Supplementary material for: Quality of antenatal care and its sociodemographic determinants: results of the 2015 Pelotas birth cohort, Brazil
Source: BMC Health Serv Res. 2021 Oct 9;21:1070. doi: 10.1186/s12913-021-07053-4 (PMC8501641; doi:10.1186/s12913-021-07053-4)
Supplement: Supplementary file 2 — Additional file 2 Supplementary file 2. Figure flowchart sample. [file 12913_2021_7053_MOESM2_ESM.docx]

**Supplementary File 2**. Figure flowchart sample

Eligible births for inclusion in the 2015 Pelotas birth cohort study

n=4329

If there were multiple pregnancies, only one record was kept for each mother. Fifty-nine records corresponded to multiple births n=59

Pelotas birth cohort mothers in 2015

n=4270

Mothers who did not attend or receive antenatal care in 2015

n=98

Pelotas birth cohort mothers that had antenatal care in 2015 n=4172
