# Supplementary material for: Quality of antenatal care and its sociodemographic determinants: results of the 2015 Pelotas birth cohort, Brazil
Source: BMC Health Serv Res. 2021 Oct 9;21:1070. doi: 10.1186/s12913-021-07053-4 (PMC8501641; doi:10.1186/s12913-021-07053-4)
Supplement: Supplementary file 3 — Additional file 3. Supplementary File 3 – Figure conceptual framework for the analysis of factors associated with the quality ANC in the 2015 Birth Cohort in Pelotas, Rio Grande do Sul, Brazil. [file 12913_2021_7053_MOESM3_ESM.docx]

Supplementary File 3 – Figure conceptual framework for the analysis of factors associated with the quality ANC in the 2015 Birth Cohort in Pelotas, Rio Grande do Sul, Brazil.

**Intermediate level**

**Distal level**

**Proximal level**

*Maternal characteristics during pregnancy*

*Health service provider characteristics*

*Maternal* *characteristics during perinatal period*

Diseases during of pregnancy (high blood pressure and/or diabetes)

-Smoking during of pregnancy

Alcohol use during of pregnancy

- Age

-Maternal education

- Marital status

- Skin color

-Family income

-Parity

- Type of health service provider

-The same professional performed ANC

Quality ANC
