# Supplementary material for: Quality of antenatal care and its sociodemographic determinants: results of the 2015 Pelotas birth cohort, Brazil
Source: BMC Health Serv Res. 2021 Oct 9;21:1070. doi: 10.1186/s12913-021-07053-4 (PMC8501641; doi:10.1186/s12913-021-07053-4)
Supplement: Supplementary file 4 — Additional file 4 Supplementary file 4. Diagrams of conceptual framework of the effects of maternal education on the quality of antenatal care received. [file 12913_2021_7053_MOESM4_ESM.docx]

**Supplementary File 4.** Diagrams of conceptual framework of the effects of maternal education on the quality of antenatal care received.

**Diagram 1.**

** Diagram 2.**
