# Supplementary material for: Quality of antenatal care and its sociodemographic determinants: results of the 2015 Pelotas birth cohort, Brazil
Source: BMC Health Serv Res. 2021 Oct 9;21:1070. doi: 10.1186/s12913-021-07053-4 (PMC8501641; doi:10.1186/s12913-021-07053-4)
Supplement: Supplementary file 5 — Additional file 5 Supplementary file 5. Frequency of the Antenatal care content quality score in categories according to the independent variables. [file 12913_2021_7053_MOESM5_ESM.docx]

| **Supplementary File 5. Frequency of the Antenatal care content quality score in categories according to the independent variables.** | | | |
| --- | --- | --- | --- |
| **Characteristics** | **Antenatal care content quality score in categories** | | |
|  | **Inadequate ≤ 15 N (%)** | **Moderate ≥16 to ≤17 N (%)** | **Adequate ≥18 to ≤19 N (%)** |
| **Age (years)** |  |  |  |
| ≤19 | 260(43.2) | 219(36.4) | 123(20.4) |
| 20-29 | 684(34.5) | 824(41.6) | 474(23.9) |
| 30-39 | 478(32.6) | 604(41.2) | 384(26.2) |
| ≥40 | 40(32.8) | 62(50.8) | 20(16.4) |
| **Maternal education (complete years of schooling)** |  |  |  |
| 0-4 | 166(45.5) | 117(32.1) | 82(24.4) |
| 5-8 | 416(39.6) | 404(38.4) | 230(22.0) |
| 9-11 | 478(33.0) | 586(40.5) | 383(26.5) |
| 12 + | 402(30.7) | 602(45.9) | 306(23.4) |
| **Marital status** |  |  |  |
| Without partner | 278(48.4) | 198(34.4) | 99(17.2) |
| With partner | 1.184(32.9) | 1.511(42.0) | 902(25.1) |
| **Skin color** |  |  |  |
| White | 998(33.8) | 1.242(42.0) | 714(24.2) |
| Black/brown | 448(37.7) | 456(38.4) | 283(23.9) |
| Other | 16(51.6) | 11(35.5) | 4(12.9) |
| **Family income (quintiles)** |  |  |  |
| Lowest/first | 333(41.0) | 283(34.8) | 197(24.2) |
| Second | 296(35.8) | 318(38.5) | 213(25.7) |
| Third | 292(34.5) | 343(40.5) | 211(25.0) |
| Fourth | 289(34.2) | 368(43.6) | 187(22.2) |
| Highest/fifth | 252(29.9) | 396(47.1) | 193(23.0) |
| **Parity** |  |  |  |
| Primiparous | 679(32.5) | 899(43.1) | 510(24.4) |
| ≥ 2 children | 782(37.5) | 810(38.9) | 491(23.6) |
| **Diseases during pregnancy (high blood pressure and/or diabetes)** |  |  |  |
| Yes | 453(35.4) | 522(40.8) | 304(23.8) |
| No | 1.009(34.9) | 1.187(41.0) | 304(24.1) |
| **Smoking during pregnancy** |  |  |  |
| Yes | 288(43.6) | 217(32.8) | 156(23.6) |
| No | 1.173(33.4) | 1.492(42.5) | 845(24.1) |
| **Alcohol use during pregnancy** |  |  |  |
| Yes | 122(40.4) | 123(40.7) | 57(18.9) |
| No | 1.339(34.6) | 1.586(41.0) | 943(24.4) |
| **Type of health care provider** |  |  |  |
| Public | 404(29.1) | 523(38.6) | 448(32.3) |
| Private | 511(29.9) | 815(47.6) | 386(22.5) |
| **The same professional performed the ANC** |  |  |  |
| Yes | 859(38.7) | 882(39.7) | 480(21.6) |
| No | 603(30.9) | 827(42.4) | 521(26.7) |
| **Total** | 1.462(35.0) | 1.709(40.9) | 1.001(24.1) |
| *The total of some variables does not sum to 4172 because of missing data. | | | |
